# Neonatal metabolic alkalosis and mild diuresis resulting from torasemide self-medication by the mother: a case report

**DOI:** 10.1186/s40780-025-00436-3

**Published:** 2025-04-11

**Authors:** Yumi Kitahiro, Mari Hashimoto, Yukako Sonda, Miki Yagi, Kotaro Itohara, Takumi Kido, Kazumichi Fujioka, Hitomi Imafuku, Tomohiro Omura, Ikuko Yano

**Affiliations:** 1 Department of Pharmacy, Kobe University Hospital, 7-5-2 Kusunoki-cho, Chuo-ku, Kobe, 650-0017 Japan; 2 Department of Pediatrics, Graduate School of Medicine, Kobe University, 7-5-2 Kusunoki-cho, Chuo-ku, Kobe, 650-0017 Japan; 3 Department of Obstetrics and Gynecology, Graduate School of Medicine, Kobe University, 7-5-2 Kusunoki-cho, Chuo-ku, Kobe, 650-0017 Japan

**Keywords:** Metabolic alkalosis, Newborn, Pregnancy, Torasemide, Loop diuretic

## Abstract

**Background:**

Torasemide, a loop diuretic, is rarely used for pregnant women because of the risk of reduced placental blood flow resulting from decreased circulating plasma volume. We experienced a case of a newborn with metabolic alkalosis and mild polyuria. The mother was suspected of self-medicating as we detected torasemide in the neonatal serum by LC-MS/MS method.

**Case presentation:**

A Japanese pregnant woman in her 20s with mental illness, symptoms of panic and eating disorders, and a history of overdosing on over-the-counter medications, was referred to our hospital for birth control. She presented with vomiting following bulimia nervosa and hypokalemia. Her baby was delivered vaginally at 36 weeks and 4 days of gestation. The baby’s blood gas analysis on day 0 revealed metabolic alkalosis (pH > 7.42, HCO_3_^-^ > 28 mmHg). Up to 16 h after birth, mild polyuria and a urine output of 3.3 mL/kg/h were observed without the administration of diuretics. We suspected diuretic intake by the mother before delivery, because she had a history of taking torasemide before being referred to the hospital. As expected, torasemide was detected in the baby’s serum. The serum concentration on the first day after delivery (4.80 ng/mL) gradually decreased to 0.45 ng/mL on day 5, whereas torasemide was not detected in the maternal serum. Neonatal metabolic alkalosis improved by day 3 following birth.

**Conclusions:**

This case suggests close counseling and monitoring of pregnant women before childbirth regarding their past and present use of drugs, particularly in those with mental illness.

## Background

Pregnant women with severe mental disorders are at higher risk for prematurity and impaired fetal development [1]. Eating disorders in women of childbearing age are not only the highest out of all age categories, but are on an increasing trajectory [2]. A previous report suggests that patients with anorexia nervosa experience hypertension, miscarriage, difficult labor, and premature delivery [3]. Kuobaa et al. [4] listed several complications associated with eating disorders in pregnancy, including low birth weight and increased risk of microcephaly. Therefore, pregnant women with mental illness must be carefully managed during pregnancy.

As a mental illness that has recently attracted attention, some females and/or young people tend to engage in self-harm using over-the-counter (OTC) medications [5]. Moreover, the Internet facilitates the availability and acquisition of OTC medications. During pregnancy, substance abuse disorder is one of the most common risk factors for pregnancy-associated suicide [6]. A significant number of pregnant women with such disorders have one or more psychiatric diagnoses, which can exacerbate the problem of substance abuse disorder [7, 8]. The most prominent example of inappropriate use of drugs is acetaminophen overdose [9]. Therefore, pregnant women with mental illness should be carefully counseled and monitored.

Diuretics are not generally used during pregnancy because of the risk of reduced placental blood flow resulting from decreased circulating plasma volume [10]. The loop diuretic furosemide has been reported to reduce the intravascular volume during the postpartum period and assist with blood pressure control and readmission rates [11]. During pregnancy, furosemide is a relatively safe drug, if used sparingly and administered effectively for the symptoms of volume overload or volume control during heart failure [12, 13]. In contrast, torasemide, another loop diuretic, has rarely been used for pregnancy because it has approximately 10-fold more potent diuretic effects compared with furosemide [14].

We experienced a case, involving a mother with panic and eating disorders as well as a history of substance abuse of OTC medications. Her baby presented with metabolic alkalosis and a mild polyuria immediately after birth. Here, we report the possibility of a torasemide self-mediating mother and confirm the presence of torasemide in her neonate’s serum using a high-performance liquid chromatography-tandem mass spectrometry (LC-MS/MS) method.

## Case presentation

The pregnant Japanese woman was in her 20s and became pregnant with her third child. The patient experienced panic disorder, an eating disorder (bulimia nervosa), and a history of abusing OTC medications. She was referred to the Kobe University Hospital for birth control at the gestational age of 25 weeks. After medical interviews, it was determined that the patient had self-medicated with torasemide due to edema during pregnancy. There was a possibility that the mother obtained torasemide by herself on the internet; however, the details of the brand and its dosage were not determined. Torasemide was substituted with a Japanese herbal medicine *Saireito* (KB-114, Kracie, Tokyo, Japan, 8.10 g/d) at approximately 34 and 35 weeks of gestation. Another drug, zolpidem, was concomitantly administered to treat insomnia. The patient presented with vomiting following bulimia nervosa and hypokalemia, and a laboratory test value just before delivery was 2.9 mmol/L. A baby weighing 2,566 g was delivered vaginally at 36 weeks and 4 days of gestation.

**Fig. 1:**
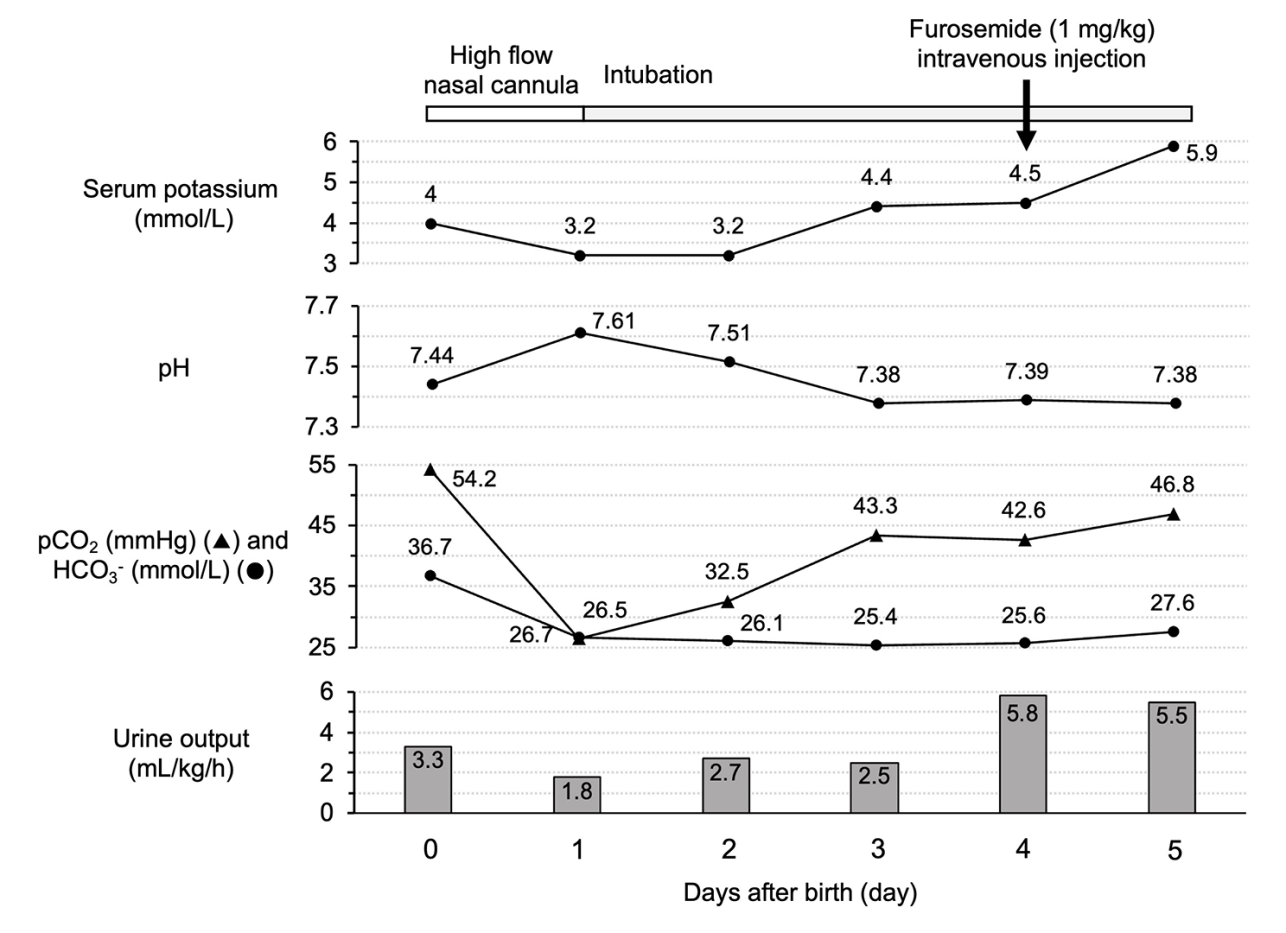
Laboratory test and physiological function data in the baby

The Apgar score for the baby was 8 and 9 points (cyanosis) at 1 and 5 min, respectively, and the blood gas analysis showed metabolic alkalosis (pH > 7.42, HCO_3_^-^ > 28 mmHg) (Fig. 1). The partial pressure of arterial carbon dioxide seemed to be increased as a compensatory reaction to metabolic alkalosis but was not measured. The baby was controlled by respiratory management in the incubator and high-flow nasal cannula. During the first hour after birth, pulses on all extremities and the heart rhythm were normal; however, the baby presented with polycythemia, and fluid replacement was administered. Up to 16 h after delivery, mild polyuria with a urine output of 3.3 mL/kg/h was observed without the administration of diuretics; although amniotic fluid volume of the patient during pregnancy had been normal. Thus, there was a suspicion that the mother was using a diuretic before delivery, because she had a history of using torasemide before consulting our hospital. Neonatal metabolic alkalosis improved within day 3 following birth. The patient provided verbal informed consent for the study, including measurement and publication of drug concentrations in her serum as well as that of her baby by routine laboratory tests.

The concentration of torasemide was determined by LC-MS/MS (LCMS-8030, Shimadzu, Kyoto, Japan). Acetonitrile (90 µL) and 10 ng/mL torasemide-d7 (Internal standard) were added to 30 µL of serum sample. After centrifugation at 24,981×g for 5 min, 6 µL of the supernatant was injected into the system. The chromatographic separation was performed on a Mastro C18 analytical column (50 × 2.1 mm, i.d.; 3 μm, Shimadzu) with mobile phase A consisting of 0.1% formic acid in water and mobile phase B consisting of 0.1% formic acid in acetonitrile. A mobile phase gradient was used with varying percentages of solvent B within A and other conditions as follows; 0 min, B 40%; 0.70 min, B 40%; 1.0 min, B 80%; 2.5 min, B 98%; 4.0 min, B 98%; 4.01 min, B 40% and hold for 1 min. The column temperature was maintained at 40 °C with a flow rate of 0.2 mL/min. The analytes were detected as follows: Positive mode: torasemide 348.75 > 263.90 and torasemide-d7 355.75 > 264.00. The serum calibration curve showed linearity with a coefficient of determination (R^2^) > 0.999 and the lowest limit of detection for torasemide at 0.15 ng/mL. The validation was performed using the quality control samples of torasemide (0.3, 1.25, and 5 ng/mL, in triplicate) with a precision ≤ 20% and intraday accuracy within 80–120% of the nominal concentration.

**Fig. 2:**
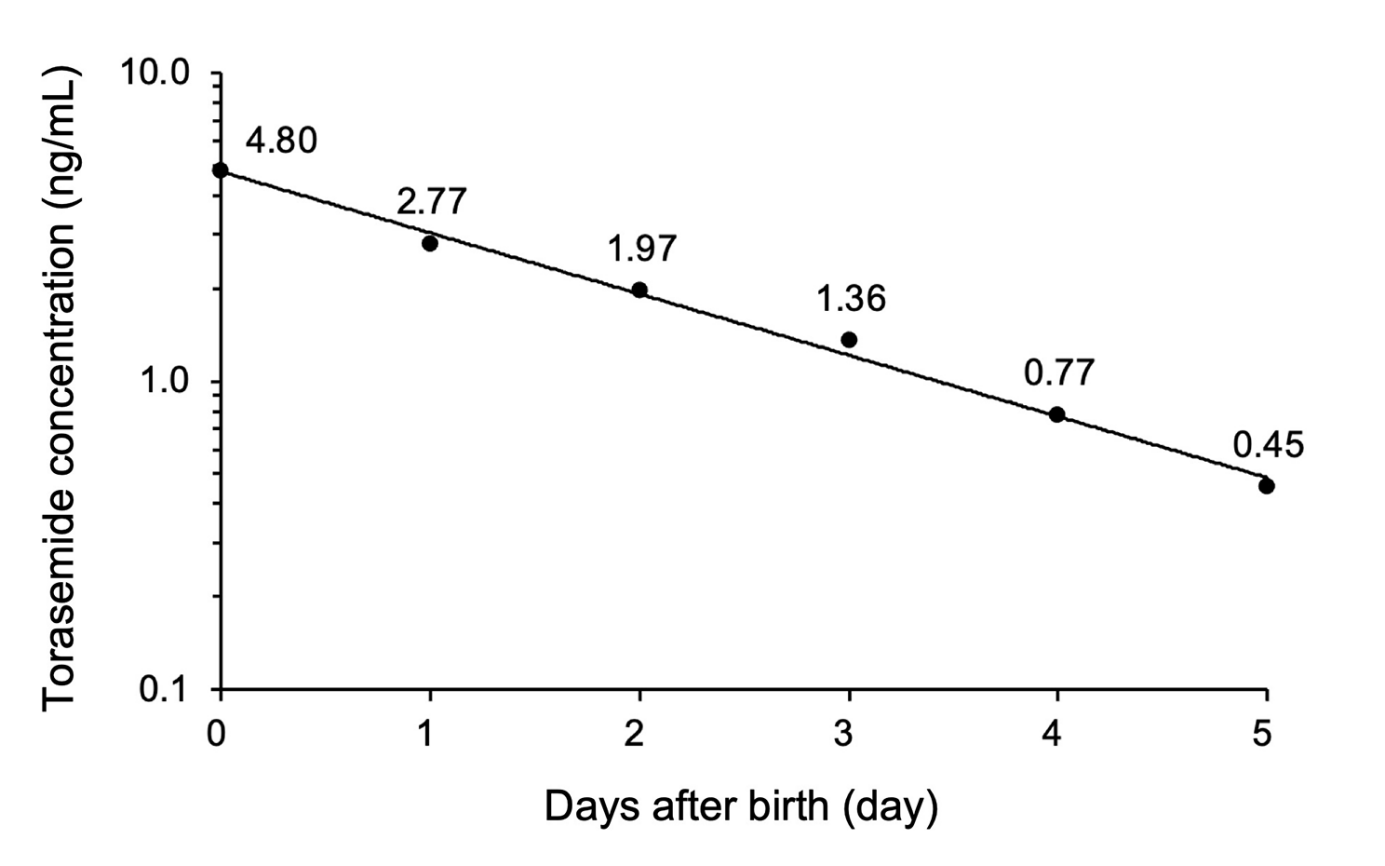
Time course of torasemide concentrations in the neonatal serum after birth

As expected, torasemide was detected in the neonatal serum (Fig. 2). The serum concentration on day 0 (4.80 ng/mL) gradually decreased to 2.77, 1.97, 1.36, 0.77, and 0.45 ng/mL on days 1 to 5, respectively. However, torasemide was not detected in the maternal serum on day 1. In addition, we attempted to detect furosemide considering the possibility that other diuretics were taken, because the pharmacist in our hospital obtained the information from the mother that she had taken furosemide before. However, furosemide was not observed in the maternal or neonatal serum.

## Discussion and conclusions

Metabolic alkalosis is characterized by an increase in serum bicarbonate and arterial pH [15]. Vomiting or eating disorders tend to cause metabolic alkalosis in pregnant women, whereas an electrolyte/acid-base disturbance in the mother is transferred to the child [16]. Pseudo-Bartter’s syndrome is a disorder, in which patients present with Bartter’s syndrome-like symptoms, such as vomiting, diarrhea, and neurogenic emaciation because of secondary factors, such as prolonged use of diuretics and laxatives. When the mother presents with hypokalemia and metabolic alkalosis, the newborn may show similar abnormalities and require neonatal resuscitation to compensate for respiratory depression [17, 18]. In the present case, the mother ingested a diet during pregnancy, and no urinary ketones were observed in laboratory tests. However, the mother had an eating disorder of bulimia nervosa and presented with vomiting, which may have affected her baby’s metabolic alkalosis.

In the present case, increased urine output in the baby and metabolic alkalosis was an uncommon finding. The mother had taken torasemide frequently using her own judgment. Concerned with edema during pregnancy, the Japanese herbal medicine *Saireito* (KB-114) was administered at 8.10 g/d at approximately 34 and 35 weeks of gestation by her physician. However, she took 24.3 g/d of *Saireito* on her own as she was concerned about heavy edema. MacGregor et al. [19] reported a transient edema following discontinuation of loop diuretics, which may be a reason why the mother complained about edema. We also considered the possibility of pseudo-hyperaldosteronism. *Saireito* is prescribed to alleviate various types of water retention and edema [20]. Glycyrrhizic acid, which is the main component of licorice (Glycyrrhizae Radix) in *Saireito*, causes pseudo-hyperaldosteronism, a clinical condition characterized by hypertension, hypokalemia, and suppression of plasma renin and aldosterone levels [21]. However, the patient did not show hypertension and her serum sodium levels were normal (the laboratory test just before delivery was 139 mmol/L), which may not have contributed to the metabolic alkalosis.

The baby’s serum anion gap (AG) on day 0, calculated as [Na^+^]– ([HCO_3_^−^] + [Cl^−^]), supports the diagnosis by classifying the disorders as a normal (hyperchloremic) anion gap or elevated anion gap [22]. The present neonate’s AG and corrected HCO_3_^-^ on day 0 was 6.3 (normal AG value ranges between 12 ± 2) and 31 (calculated as [HCO_3_^-^] + ΔAG and normal corrected HCO_3_^-^ value ranges between 24 and 26 mmol/L), respectively, showing renal tubular alkalosis [15]. Therefore, the baby’s metabolic alkalosis was caused not only by vomiting of the mother, but also other causes, and the suspicion of diuretic use before delivery was suspected following the patient’s medical history.

Torasemide is primarily metabolized by the hepatic cytochrome P450 (CYP) 2C9 enzyme [23, 24], with a half-life (t_1/2_) of 2.0 ± 0.8 h after repeated administration [25]. In addition, the excretion of metabolites and torasemide in urine is 50–80% [25]. In the present case, the neonatal serum concentration on day 0 gradually decreased. The expression levels and activity of CYP2C9 in neonates are higher compared with those of other CYP species [26]. The rapid decrease in the serum concentrations of torasemide in the baby suggests that CYP2C9 may contribute to the metabolism of torasemide. Whereas, torasemide was not detected in the maternal serum despite its detection in the neonate serum. The concentration of torasemide in the neonatal plasma was reported to be one-fifteenth of that in the maternal plasma in a rat model [27]. In the present case, the last intake time and dose of torasemide were not provided by the mother. On one hand, there is an increase in the activity of CYP2C9 and CYP3A4, hepatic blood flow, renal blood flow, and circulating blood volume during pregnancy [28, 29]. In addition, because torasemide has a short t_1/2_, it may be eliminated rapidly and below the lower detection limit in the mother.

This case study had some limitations. First, precise medication information was not obtained from the mother. There was a possibility that the mother obtained torasemide by herself on the internet; however, the details of the brand and its dosage were not determined. Second, the maternal serum sample represented only one point and we did not know the time course of torasemide concentration. This study included only one maternal case and her baby, thus further information on torasemide in perinatal women is needed.

Substance abuse through self-administration can result in significant toxicity in pregnant women and neonates [30]. Because of the multiple clinical and psychosocial challenges of preventing pregnancy-related overdose, care for women at risk requires a multipronged approach. Therefore, close counseling and monitoring of pregnant women concerning the past and present use of drugs should be performed, particularly in mothers with mental illness.

## Data Availability

No datasets were generated or analysed during the current study.

## Abbreviations

AG: anion gap
CYP: hepatic cytochrome P450
OTC: over-the-counter
t_1/2_: half-life
